# Phenotype-informed selection of microbiota-directed interventions in irritable bowel syndrome: current evidence and limitations

**DOI:** 10.3389/fphar.2026.1921349

**Published:** 2026-10-13

**Authors:** Om Kolthoum Sallem

**Affiliations:** Department of Nutrition, Faculty of Medicine of Monastir, EPS Fattouma Bourguiba, Monastir, Tunisia

**Keywords:** fecal microbiota transplantation, gut microbiota, irritable bowel syndrome, phenotype-informed care, postbiotics, prebiotics, probiotics, rifaximin

## Abstract

Irritable bowel syndrome (IBS) is a heterogeneous disorder of gut–brain interaction in which bowel-habit subtype, dominant symptoms, post-infectious onset, bile-acid handling, visceral sensitivity, and diet–microbiota interactions may influence the treatment response. This narrative mini-review asks whether available clinical evidence is sufficient to select or sequence microbiota-directed and microbiota-modulating interventions according to Rome bowel-habit subtype and clinically observable dominant symptoms. Literature was identified through PubMed/MEDLINE and major guidelines, systematic reviews, meta-analyses, and pivotal trials published through June 2026. Evidence was interpreted at the level of the exact intervention or product, studied population, target outcome, consistency, independent replication, safety, and guideline position. Mechanistic data support plausible links through fermentation, epithelial barrier function, mucosal immunity, bile-acid metabolism, visceral sensitivity, and gut–brain signaling, but mechanistic or microbiome changes do not establish the clinical efficacy. Rifaximin has the clearest replicated and guideline-supported role in IBS with diarrhea, while soluble fiber has guideline support for global or constipation-associated symptoms. Evidence for individual probiotics, postbiotics, partially hydrolyzed guar gum, galactooligosaccharides, and fermented matrices remains product-specific or emerging and is rarely independently replicated. Symptom improvement with a diet low in fermentable oligosaccharides, disaccharides, monosaccharides, and polyols (low-FODMAP diet) does not establish microbiota mediation. Fecal microbiota transplantation (FMT) and microbiome-guided prescribing remain investigational. Current evidence, therefore, supports only limited phenotype-informed matching and does not establish a validated treatment sequence or microbiome-defined therapeutic phenotype.

## Introduction

Irritable bowel syndrome (IBS) is defined by recurrent abdominal pain associated with defecation and changes in stool frequency or form after appropriate clinical evaluation and exclusion of relevant organic disease (Mearin et al., 2016). Clinical heterogeneity complicates treatment selection because bowel-habit subtype, dominant symptoms, post-infectious onset, bile-acid handling, diet, and gut–brain factors may create different therapeutic priorities.

Gut microbiota is biologically relevant to IBS because microbial metabolism and host–microbe signaling can influence fermentation, intestinal transit, epithelial permeability, mucosal immune activity, bile-acid transformation, visceral sensitivity, and neural signaling (Xiao et al., 2021; Barbara et al., 2019; Duboc et al., 2012; Tap et al., 2017; Pittayanon et al., 2019; Li et al., 2024). These observations support therapeutic investigation but do not establish dysbiosis as a clinical diagnosis or justify routine microbiome-guided prescribing.

Several recent syntheses, including those by Li et al. (2025), have already reviewed dysbiosis, candidate mechanisms, and the principal microbiome-based therapies in IBS. The purpose of this mini-review is, therefore, not to recatalog these interventions. It investigates whether available clinical evidence is sufficient to select or sequence them according to Rome bowel-habit subtype and clinically observable dominant symptoms.

Throughout this review, IBS with diarrhea (IBS-D), IBS with constipation (IBS-C), IBS with mixed bowel habits (IBS-M), and unclassified IBS (IBS-U) denote Rome bowel-habit subtypes. Dominant symptoms such as bloating, gas, or pain are described as symptom profiles rather than separate IBS phenotypes. The term non-constipated IBS is retained only when reporting the population enrolled in the pivotal rifaximin trials. Post-infectious onset and suspected bile-acid diarrhea are treated as clinical modifiers rather than Rome subtypes. Fermentable oligosaccharides, disaccharides, monosaccharides, and polyols are abbreviated as FODMAPs.

## Methods

This article was designed as a focused narrative mini-review rather than a systematic review or meta-analysis. The review question was whether currently available evidence is sufficient to support phenotype-informed selection or sequencing of microbiota-directed and microbiota-modulating interventions in adults with IBS.

Search strategy and source selection. Literature identification was performed through an iterative search of PubMed/MEDLINE and the reference lists of major clinical guidelines, consensus statements, systematic reviews, meta-analyses, and pivotal randomized controlled trials published up to June 2026. The search terms included combinations of IBS, IBS-D, IBS-C, IBS-M, gut microbiota, microbiome, dysbiosis, rifaximin, probiotic, postbiotic, prebiotic, synbiotic, psyllium, ispaghula, partially hydrolyzed guar gum, fermented milk, *Bifidobacterium*, *Lactiplantibacillus*, *Lacticaseibacillus*, *Weizmannia coagulans*, fecal microbiota transplantation (FMT), bile acids, low-FODMAP diet, guideline, randomized trial, systematic review, and meta-analysis. Reference lists were checked for additional clinically relevant studies.

Eligibility and data extraction. Eligible publications included adult IBS trials, systematic reviews, meta-analyses, clinical practice guidelines, consensus statements, and human mechanistic studies directly relevant to symptom management. Pediatric-only, animal-only, *in vitro*-only, anecdotal, or non-IBS organic disease studies were excluded unless they directly informed mechanism or safety. Observational microbiome studies were retained only when they clarified biological plausibility or candidate stratification signals and were not interpreted as diagnostic or treatment-selection tools. For each intervention, we extracted the studied IBS subtype or symptom profile, intervention identity, dose or substrate, duration, comparator, target outcome, assessment point, adverse events, durability or retreatment information, and guideline position when available.

Evidence synthesis and clinical framework. Evidence was interpreted according to the exact intervention, studied population, target outcome, consistency, independent replication, safety, and guideline position. Each intervention-phenotype link was categorized as guideline-supported or replicated, product-specific or emerging, or mechanistically plausible or experimental. These categories are qualitative judgments for this narrative review and are not formal Grading of Recommendations Assessment, Development and Evaluation (GRADE) ratings. The clinical framework was developed by mapping each intervention to the population, target symptom, dose, duration, and outcome studied. It is an author-proposed, evidence-informed framework and has not been prospectively validated.

Because the treatment duration differs substantially among rifaximin, fibers, probiotics, postbiotics, prebiotics, and dietary strategies, no universal follow-up threshold was imposed. Before treatment, the target symptom, baseline measure, regimen, expected duration, and assessment point should be defined from the relevant trial or guideline. Benefit and tolerability should be reviewed at the end of that planned window, with earlier discontinuation for adverse events, clear symptom worsening, or diagnostic red flags. These discontinuation and reconsideration criteria are pragmatic safeguards, not validated IBS-specific efficacy thresholds.

Methodological limitations. No formal risk-of-bias scoring, duplicate independent screening, quantitative pooling, or formal GRADE assessment was performed. Accordingly, this review emphasizes evidence calibration and transferability rather than numerical certainty estimates.

### Biological rationale and limits of microbiota as a therapeutic target

Microbial functions may intersect with IBS at several linked levels. Fermentation of dietary substrates determines gas production, osmotic load, and short-chain fatty acid profiles, which can influence luminal distension, secretion, transit, and epithelial function (Xiao et al., 2021). Increased epithelial permeability in some patient subsets may increase mucosal exposure to microbial products and contribute to low-grade immune activation, including mast-cell and cytokine signaling near enteric nerves. These events may lower sensory thresholds and amplify visceral pain, although they are not universal features of IBS.

Microbial metabolism also modifies the bile-acid pool. Reduced conversion of primary to secondary bile acids or excess delivery of bile acids to the colon may promote secretion and accelerated transit in a subset of patients with IBS-D (Duboc et al., 2012). Other microbial metabolites, including short-chain fatty acids and tryptophan-derived products, may signal through enteroendocrine, immune, vagal, and spinal pathways relevant to visceral sensitivity and gut–brain communication (Xiao et al., 2021). These mechanisms overlap and may vary with bowel-habit subtype, diet, medication exposure, and post-infectious onset (Barbara et al., 2019).

The interventions considered in this review act at different levels of this system. Rifaximin modifies luminal microbial activity; soluble fibers and prebiotics alter substrate availability and fermentation; probiotics and postbiotics deliver defined organisms or microbial components; dietary strategies change the ecological substrate; and FMT attempts ecosystem-level transfer. This mapping provides biological hypotheses, not proof of benefit. A change in taxa, metabolites, permeability markers, or immune mediators is not considered sufficient for a clinical recommendation unless a defined intervention produces reproducible improvement in patient-important outcomes in the relevant population.

Microbiome studies repeatedly identify group-level differences in IBS, but the direction and magnitude of these signals vary by sampling site, geography, diagnostic criteria, bowel-habit subtype, diet, and medication exposure (Duboc et al., 2012; Tap et al., 2017; Pittayanon et al., 2019; Li et al., 2024). No microbial signature has been validated to select a treatment for an individual patient. Consequently, commercial stool microbiome reports should not be used as a stand-alone diagnostic or prescribing tool.

### Clinically actionable phenotype information: what can and cannot be used

The most clinically defensible stratifiers are conventional ones: Rome bowel-habit subtype, dominant symptom burden, prior treatment response, medication exposure, post-infectious onset, suspected bile-acid diarrhea, diet-related symptom patterns, safety risks, and patient preference. These variables are observable and can be aligned with the population studied in a trial or addressed by a guideline.

In contrast, a putative dysbiosis pattern, a single taxon, or an unvalidated commercial microbiome score does not define an actionable therapeutic phenotype. A microbiota-directed intervention should, therefore, be considered only when its evidence base maps to the patient’s clinical context and the target outcome is explicit.

### Where phenotype-informed matching is best supported

#### Rifaximin: replicated and guideline-supported in IBS with diarrhea

Rifaximin remains the clearest microbiota-directed pharmacological intervention. In pivotal randomized trials, a 14-day course improved global IBS symptoms and bloating in enrolled patients with non-constipated IBS (Pimentel et al., 2011). Repeat-treatment data later supported benefit and safety in patients with recurrent IBS-D who had responded to an initial course (Lembo et al., 2016). The American College of Gastroenterology (ACG) guideline supports use of rifaximin for alleviating global IBS-D symptoms (Lacy et al., 2021).

This evidence should not be extrapolated to a generic dysbiosis indication. The strongest clinical match is an appropriately evaluated patient with IBS-D, particularly when diarrhea and bloating are prominent. The term non-constipated IBS describes the older trial eligibility population and should not be used as a synonym for IBS-D. The response should be assessed after the studied 14-day regimen; retreatment is relevant after recurrence in previous responders and should account for prior response, cost, local availability, and antimicrobial stewardship (Lembo et al., 2016; Lacy et al., 2021).

#### Soluble fiber: guideline-supported for global and constipation-associated symptoms

Soluble fiber has a stronger and more transferable evidence base than many microbiota-targeted products. Psyllium/ispaghula improved adequate symptom relief in a primary-care randomized trial and is supported in contemporary IBS guidance, particularly for global symptoms and constipation-associated presentations (Lacy et al., 2021; Bijkerk et al., 2009; Vasant et al., 2021; Choi et al., 2025; National Institute for Health and Care Excellence, 2017). Its benefit should not be interpreted as evidence that microbiota change mediates the clinical response; physicochemical effects on stool form, water-holding capacity, and fermentation are also relevant.

### Product-specific or emerging evidence

#### Probiotics and postbiotics: no transferable class effect

Across recent meta-analyses, probiotics may improve global symptoms, abdominal pain, or bloating, but confidence in a class effect remains low or very low (Goodoory et al., 2023; Chen et al., 2023; Xie et al., 2023; Konstantis et al., 2023; Ballena-Caicedo et al., 2026). Trials pool biologically non-equivalent organisms and mixtures, use different doses and matrices, enroll heterogeneous IBS populations, and apply nonuniform endpoints and assessment windows. Statistical benefit in pooled analyses, therefore, does not identify which product should be prescribed to which patient. Negative or null trials and outcome-specific discordance further limit reproducibility.

Evidence for *Bifidobacterium longum* 35624 (formerly reported as *Bifidobacterium infantis* 35624) includes a pivotal randomized trial and supportive real-world observations, but the latter do not provide blinded independent replication (Whorwell et al., 2006; Sabate and Iglicki, 2022). Evidence for *Lactiplantibacillus plantarum* 299v DSM 9843 is not fully consistent across studies (Ducrotte et al., 2012). Viable *Bifidobacterium bifidum* MIMBb75 and heat-inactivated *B. bifidum* HI-MIMBb75 must be considered separate interventions (Guglielmetti et al., 2011; Andresen et al., 2020). Recent studies of *B. longum* CECT 7347 ES1 or HT-ES1, *Bifidobacterium animalis subsp. lactis* BI040, *W. coagulans* BC300 (formerly *Bacillus coagulans*), and *Lacticaseibacillus rhamnosus* IDCC 3201 remain single-product signals requiring independent replication (Srivastava et al., 2024; Skrzydlo-Radomanska et al., 2023; Kwon et al., 2024). None establishes a transferable class effect.

Guidelines apply different thresholds to this uncertain evidence. The American College of Gastroenterology (ACG) advises against using routine probiotics for alleviating global IBS symptoms because of very low-certainty evidence, while the American Gastroenterological Association (AGA) restricts their use in IBS to the context of a clinical trial (Lacy et al., 2021; Su et al., 2020). Guidance from the British Society of Gastroenterology and the National Institute for Health and Care Excellence (NICE) permits a monitored, time-limited trial of a defined product but do not establish a class effect or endorse a sequence among the products reviewed here (Vasant et al., 2021; National Institute for Health and Care Excellence, 2017). The clinically defensible conclusion, consistent with product-specific guidance (Guarner et al., 2024), is, therefore, an optional, time-limited trial of a defined product with an explicit target and pragmatic stopping criteria for lack of benefit or intolerance, not routine class-level prescribing. These criteria are safeguards rather than validated IBS-specific thresholds.

#### Prebiotic substrates, partially hydrolyzed guar gum, synbiotics, and fermented matrices

Partially hydrolyzed guar gum (PHGG) has randomized evidence for its efficacy in alleviating bloating and gas-related symptoms (Niv et al., 2016). Galactooligosaccharide substrates can increase fecal bifidobacteria and may improve selected symptoms, but the evidence is sparse and dose-sensitive, and greater fermentability may worsen symptoms in patients with marked FODMAP sensitivity (Silk et al., 2009; Wilson et al., 2019). These findings are product- or substrate-specific and should not be extrapolated to all prebiotics.

Synbiotic and fermented-matrix studies require the same discipline. A fermented milk containing *B. animalis subsp. lactis* DN-173 010/CNCM I-2494 improved abdominal distension and transit in IBS-C, but this does not establish its efficacy for all fermented foods or all *B. animalis subsp. lactis* strains (Agrawal et al., 2009). Recent findings for *Lacticaseibacillus rhamnosus* IDCC 3201 in IBS-C are promising but require independent replication (Kwon et al., 2024). Microbiota or metabolite changes are supportive mechanistic outcomes; they do not substitute for reproducible patient-reported clinical benefit.

### Contextual co-interventions rather than microbiota-specific prescriptions

#### Low-FODMAP dietary care

Structured low-FODMAP dietary care can improve symptoms in selected patients and is included in contemporary IBS guidance (Vasant et al., 2021; Choi et al., 2025; National Institute for Health and Care Excellence, 2017). However, symptom improvement does not establish that microbiota modification is the causal mediator. Low-FODMAP care is, therefore, best framed as a symptom-directed dietary co-intervention, preferably with structured reintroduction and personalization, rather than as proof of microbiota-directed treatment efficacy.

Importantly, a symptomatic response to low-FODMAP dietary care should not be interpreted as proof that dysbiosis is the causal lesion or that microbiota alteration mediates the clinical benefit. Restricting fermentable carbohydrates can reduce substrate delivery for rapid fermentation and lessen luminal distension-related stimuli, while symptom improvement may also reflect non-microbial effects on intestinal water handling and meal composition. Microbial taxonomic or metabolite changes observed during restriction may, therefore, be parallel consequences of altered substrate exposure rather than the causal pathway of symptom relief. Demonstrating microbiota mediation would require evidence that a specific microbial change lies on the causal pathway between the dietary intervention and the patient-important outcome, rather than merely co-occurring with response. In the absence of such mediation evidence, low-FODMAP care should be regarded as symptom-directed dietary therapy with structured reintroduction and personalization, not as a diagnostic test for dysbiosis or proof of a microbiota mechanism (Xiao et al., 2021; Vasant et al., 2021; Choi et al., 2025; National Institute for Health and Care Excellence, 2017).

#### Bile-acid-related considerations

Altered bile-acid profiles have been reported in subsets of IBS-D and provide a plausible host–microbe pathway linking microbial transformation to secretion and transit (Duboc et al., 2012). Suspected bile-acid diarrhea should be considered clinically in the differential diagnosis and management of diarrhea, but treatment decisions should not be based on stool microbiome taxonomy alone (Duboc et al., 2012; Lacy et al., 2021; Vasant et al., 2021). This is a clinical modifier, not a microbiome-defined IBS subtype.

### Experimental approaches and evidence gaps

#### Fecal microbiota transplantation and microbiome-guided selection

FMT is transformative for recurrent *Clostridioides difficile* infection, but IBS lacks a single pathogen, a stable microbial deficiency, or a validated donor-response model. Meta-analysis of randomized trials has not shown consistent durable global benefit, and protocols vary in donor selection, route, dose, placebo design, and outcome measurement (Wang et al., 2024). FMT should, therefore, remain investigational in IBS and be restricted to ethically approved research.

Routine commercial microbiome testing is similarly premature. Current signatures are group-level associations and do not identify which probiotic, prebiotic, dietary strategy, or FMT donor should be selected for an individual patient (Li et al., 2024). Microbiome-guided prescribing should, therefore, be considered experimental until prospective validation demonstrates clinical utility beyond standard assessment.

### Evidence-calibrated patient selection and monitoring

Patient selection is the operational bridge between microbiome science and safe clinical use. A practical approach begins with diagnostic confirmation and assessment for alarm features, followed by definition of the Rome subtype, dominant symptoms, relevant modifiers, previous treatment response, cost and access, and intervention-specific safety risks. Education, reassurance, diet review, physical activity, sleep, and stress management remain foundational because microbiota-directed options are adjuncts rather than replacements for comprehensive IBS care. The proposed evidence-calibrated workflow is summarized in Figure 1. Practical patient-selection criteria are outlined in Table 1, and the intervention-specific evidence and limitations are appraised in Table 2.

**FIGURE 1:**
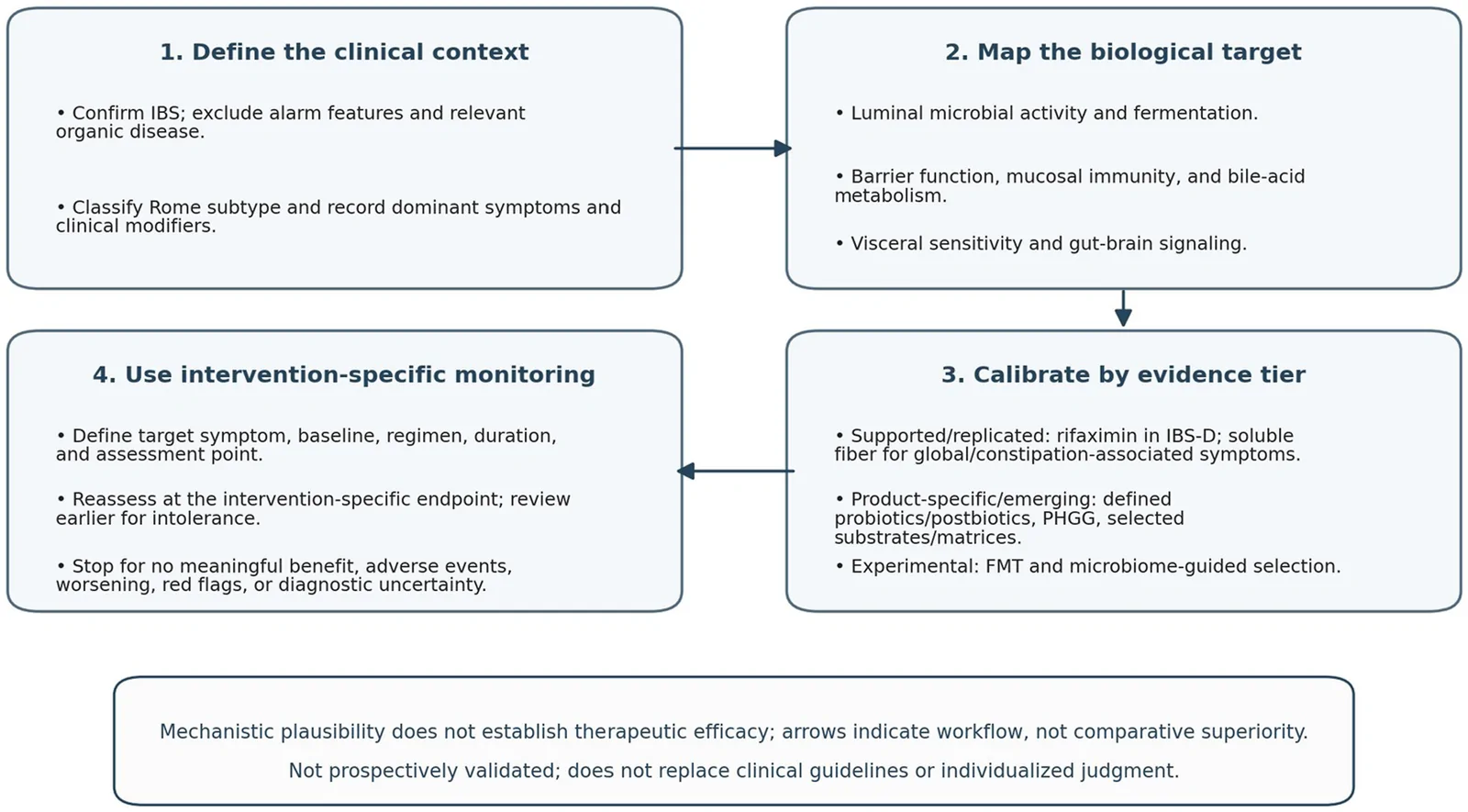
Author-proposed framework for evidence-calibrated use of microbiota-directed interventions in IBS. This framework summarizes possible clinical use and has not been prospectively validated. Arrows indicate workflow rather than comparative superiority. Guideline-supported or replicated options, product-specific or emerging options, and experimental or plausibility-based approaches are identified separately. Treatment duration and reassessment should follow the studied regimen or relevant guideline rather than a universal fixed follow-up threshold. Abbreviations: FMT, fecal microbiota transplantation; IBS, irritable bowel syndrome; IBS-C, IBS with constipation; IBS-D, IBS with diarrhea; IBS-M, IBS with mixed bowel habits; IBS-U, unclassified IBS; PHGG, partially hydrolyzed guar gum. The stopping and reconsideration criteria shown are pragmatic safeguards rather than validated IBS-specific thresholds.

**TABLE 1:** Practical patient-selection criteria for microbiota-directed or microbiota-modulating interventions in IBS

Clinical step	Select/prioritize	Avoid/reconsider
Diagnosis and safety	Rome-based IBS after appropriate evaluation; no alarm features; relevant organic disease excluded when indicated	Unexplained weight loss, rectal bleeding, iron deficiency anemia, fever, nocturnal symptoms, abnormal inflammatory markers, or other features requiring further evaluation
Subtype and symptom profile	Define IBS-D, IBS-C, IBS-M, or IBS-U; record dominant target symptoms such as diarrhea, constipation, bloating/gas, or pain. Record post-infectious onset and suspected bile-acid diarrhea as modifiers	Unspecified “dysbiosis” as the only indication; treatment selected solely from a commercial stool microbiome report
Evidence-calibrated matching	Rifaximin for appropriately selected IBS-D; soluble fiber for global or constipation-associated symptoms; product-specific trials only when direct evidence matches the target; low-FODMAP care as a structured co-intervention	Class-level probiotic prescribing; empirical multi-product combinations; extrapolating one strain, substrate, or fermented matrix to an entire class; FMT outside approved research
Safety screening	Medication and comorbidity review; intervention-specific contraindications; clear adverse-event counseling and capacity for follow-up	Use of live microbial products in severe immunosuppression, critical illness, central venous catheterization, active bloodstream infection, short bowel with translocation risk, or the inability to monitor adverse events
Monitoring and stopping	Document the baseline target, dose/regimen, duration, and the assessment point supported by the trial or guideline. Review earlier if intolerance occurs; continue only with clinically meaningful benefit	Universal fixed follow-up checkpoints unrelated to the studied regimen. Stop or reconsider for no meaningful benefit, worsening symptoms, adverse events, excessive burden/cost, red flags, or diagnostic uncertainty

Abbreviations: FMT, fecal microbiota transplantation; FODMAP, fermentable oligosaccharides, disaccharides, monosaccharides and polyols; IBS, irritable bowel syndrome; IBS-C, IBS with constipation; IBS-D, IBS with diarrhea; IBS-M, IBS with mixed bowel habits; IBS-U, unclassified IBS. The stopping and reconsideration criteria are pragmatic safeguards rather than validated IBS-specific thresholds.

**TABLE 2:** Critical appraisal of clinically relevant microbiota-directed and microbiota-modulating interventions in IBS.

Intervention or exact product	Studied IBS subtype/dominant symptom	Evidence base	Qualitative certainty and consistency	Major limitations	Guideline position/clinical status
Rifaximin	IBS-D; pivotal trials enrolled non-constipated IBS; diarrhea/bloating	Pivotal randomized controlled trials; 550 mg three times daily for 14 days; repeat-treatment trial (Pimentel et al., 2011; Lembo et al., 2016)	Guideline-supported/replicated; relatively consistent	Older “non-constipated IBS” trial population is not identical to those with Rome IBS-D; stewardship, access, and recurrence context matter	Supported by the American College of Gastroenterology for IBS-D; assess after the studied regimen; retreatment after recurrence in prior responders (Lacy et al., 2021)
Psyllium/ispaghula	Global IBS symptoms; IBS-C- or constipation-associated symptoms	Primary-care placebo-controlled RCT; guideline evidence (Bijkerk et al., 2009)	Guideline-supported; transferable at class level only to soluble fiber, not all fiber	Benefit is not proof of microbiota mediation; tolerability and titration matter	Supported in IBS guidance for global/constipation-associated symptoms (Lacy et al., 2021; Vasant et al., 2021; National Institute for Health and Care Excellence, 2017)
*Bifidobacterium longum* 35624	Global symptoms, pain, and bloating	Pivotal RCT plus supportive real-world observations (Whorwell et al., 2006; Sabate and Iglicki, 2022)	Product-specific/emerging; incomplete independent replication	Real-world data are not blinded confirmation; cannot extrapolate to other bifidobacteria	Optional product-specific trial; not a class recommendation
*Lactiplantibacillus plantarum* 299v DSM 9843	Pain and bloating/global symptom relief	Four-week RCT with later inconsistency noted (Ducrotte et al., 2012)	Product-specific; inconsistent	Limited replication and nonuniform endpoints	No class-level guideline endorsement
*Bifidobacterium bifidum* MIMBb75 and heat-inactivated HI-MIMBb75	Global IBS symptoms and quality of life	Separate randomized studies of viable and heat-inactivated preparations (Guglielmetti et al., 2011; Andresen et al., 2020)	Product-specific/emerging	Viable probiotic and heat-inactivated postbiotic are distinct interventions	May be considered only as separate product-specific evidence; no class effect
*B. longum* CECT 7347 ES1/HT-ES1	IBS-D	Randomized double-blind placebo-controlled trial (Srivastava et al., 2024)	Emerging single-product signal	Independent replication required; live and heat-treated forms must not be conflated	Investigational/product-specific rather than guideline-established
*B. animalis subsp. lactis* BI040/*Weizmannia coagulans* BC300	Symptom-specific signals across adult IBS	Three-arm randomized trial (Skrzydlo-Radomanska et al., 2023)	Emerging single-product signals	Different symptom effects; one trial; no transferable class effect	Product-specific evidence only
PHGG	Prominent bloating/gas; may coexist with constipation	Randomized placebo-controlled study (Niv et al., 2016)	Product-specific/emerging	Evidence limited relative to psyllium; gradual titration and tolerance matter	Reasonable monitored substrate trial when the target matches the study population
Galactooligosaccharide prebiotic substrates	Selected IBS symptoms; fermentation-sensitive patients require caution	Human trial plus systematic review (Silk et al., 2009; Wilson et al., 2019)	Emerging; sparse and dose-sensitive	Microbiota enrichment does not prove clinical efficacy; fermentability may worsen symptoms	Selected use only; not a class-level recommendation
Fermented milk with *B. animalis subsp. lactis* DN-173 010/CNCM I-2494	IBS-C with abdominal distension	IBS-C randomized trial (Agrawal et al., 2009)	Product/matrix-specific/emerging	Effect cannot be generalized to all fermented foods or *B. animalis* strains	No class-level recommendation
*Lacticaseibacillus rhamnosus* IDCC 3201	IBS-C	Recent randomized double-blind placebo-controlled trial (Kwon et al., 2024)	Emerging single-product signal	Independent replication required; microbiota/metabolite changes are intermediate outcomes	Product-specific; insufficient for strong recommendation
Low-FODMAP dietary care	Diet-sensitive symptoms across Rome subtypes	Guideline-supported symptom-directed dietary strategy (Vasant et al., 2021; National Institute for Health and Care Excellence, 2017)	Guideline-supported for symptom care; microbiota mediation unproven	Requires structured reintroduction/personalization; not pharmacotherapy and not proof of microbiota mechanism	Use as contextual co-intervention, preferably dietitian-supported
Fecal microbiota transplantation (FMT)	IBS across subtypes	Meta-analysis of randomized trials (Wang et al., 2024)	Experimental; inconsistent	Donor, route, dose, placebo, and outcome heterogeneity; no validated donor-response model	Research-only in IBS
Commercial microbiome-guided selection	No validated individual treatment phenotype	Cross-cohort and observational signatures (Pittayanon et al., 2019; Li et al., 2024)	Experimental/unsupported for prescribing	Group-level associations; no prospective clinical utility for selecting interventions	Do not use routinely for diagnosis or treatment selection

Certainty and consistency are qualitative judgments for this narrative review and are not formal Grading of Recommendations Assessment, Development and Evaluation (GRADE) ratings. Abbreviations: FMT, fecal microbiota transplantation; FODMAP, fermentable oligosaccharides, disaccharides, monosaccharides and polyols; IBS, irritable bowel syndrome; IBS-C, IBS with constipation; IBS-D, IBS with diarrhea; PHGG, partially hydrolyzed guar gum.

Before initiating an intervention, the clinician should document a measurable target symptom, baseline severity, the exact drug, product, or substrate, dose, planned duration, expected benefit, and adverse event plan. Rifaximin should follow its evidence-based 14-day regimen, with the response assessed after treatment and retreatment considered after recurrence in prior responders (Pimentel et al., 2011; Lembo et al., 2016). Probiotics, postbiotics, fibers, and prebiotic substrates should be assessed at the end of the product- or guideline-specific trial period, commonly 4–12 weeks as mentioned in the reviewed literature and guidance, without treating that range as a universal efficacy threshold. Earlier review is appropriate for intolerance, adverse events, or clear symptom worsening.

## Discussion and future directions

The central finding of this review is that current evidence supports phenotype-informed use only to a limited extent. Rifaximin has replicated trial and guideline support in IBS-D, and soluble fiber has guideline support for global or constipation-associated symptoms. Beyond these anchors, the evidence becomes substantially more product-specific, less consistently replicated, and more difficult to translate into a general prescribing rule.

This distinction is particularly important for probiotics and postbiotics. Pooled meta-analyses can detect average symptom benefit while remaining unable to identify a transferable class effect because the constituent trials study different organisms, combinations, doses, matrices, populations, and endpoints (Goodoory et al., 2023; Chen et al., 2023; Xie et al., 2023; Konstantis et al., 2023; Ballena-Caicedo et al., 2026). The guideline disagreement reflects different thresholds for clinical action rather than proof that all probiotic products are either effective or ineffective. ACG and AGA emphasize the low certainty and, therefore, do not support routine class-level use, whereas British and NICE guidance permits a monitored, time-limited trial of a defined product (Lacy et al., 2021; Su et al., 2020; Vasant et al., 2021; National Institute for Health and Care Excellence, 2017). These positions do not justify a sequence among individual products.

The biological rationale also requires calibration. Fermentation, epithelial barrier function, mucosal immunity, bile-acid metabolism, visceral sensitivity, and gut–brain signaling provide plausible targets, but the presence of a mechanistic signal does not establish treatment efficacy. Conversely, a clinically effective intervention need not demonstrate that microbiota change is the mediator of benefit. This is particularly relevant to soluble fiber and low-FODMAP dietary care, where symptom improvement should not be re-labeled as microbiome-specific efficacy without mediation evidence.

The author-proposed framework is, therefore, best viewed as an evidence map rather than a validated sequencing pathway. Its arrows describe a possible clinical workflow: confirm diagnosis and safety, define Rome subtype and dominant symptoms, match only to evidence that is transferable to that context, and reassess over the duration supported by the relevant trial or guideline. It does not establish that one microbiota-directed intervention should precede another or justify universal fixed follow-up checkpoints.

Future trials should test the phenotype hypothesis directly. Priorities include head-to-head comparisons, responder-enriched designs, bowel-habit subtype and dominant symptoms defined *a priori*, explicit diet and medication reporting, and consistent patient-reported outcomes. Candidate biomarkers such as bile-acid profiles, fermentation markers, metabolomics, permeability indices, and microbial features should be evaluated prospectively for treatment-effect modification rather than reported only as correlates. Comparative sequencing trials are especially needed before an individualized pathway can be described as evidence-established.

## Conclusion

Current evidence supports only limited phenotype-informed use of microbiota-directed and microbiota-modulating interventions in IBS. Rifaximin has the clearest replicated and guideline-supported role in IBS-D, and soluble fiber is supported for global or constipation-associated symptoms. Evidence for individual probiotics, postbiotics, PHGG, galactooligosaccharides, synbiotics, and fermented matrices remains product-specific or emerging and should not be extrapolated to a class. Low-FODMAP care is a symptom-directed dietary co-intervention without proof that microbiota change mediates benefit. FMT and microbiome-guided selection remain investigational. The proposed framework is an author-derived evidence map, not a validated treatment sequence; treatment duration and reassessment should follow the studied regimen or relevant guideline, with an explicit treatment target and pragmatic safety, tolerability, and lack-of-benefit safeguards rather than fixed IBS-specific thresholds.
